# RAS pathway activity subtypes identified by machine learning define prognostic and immune microenvironment characteristics in lung adenocarcinoma

**DOI:** 10.1007/s12672-026-05282-9

**Published:** 2026-05-28

**Authors:** Yao shi

**Affiliations:** Department of Respiratory and Critical Care Medicine, The Third People’s Hospital of Chengdu, Chengdu, 610014 China

**Keywords:** Lung adenocarcinoma, RAS pathway, Machine learning, Immune microenvironment, Prognostic model

## Abstract

**Background:**

Lung adenocarcinoma (LUAD), the predominant histological subtype of non-small cell lung cancer, remains a leading cause of cancer-related mortality worldwide. The RAS signaling pathway plays a critical role in LUAD pathogenesis; however, the heterogeneity of RAS pathway activity and its clinical implications remain poorly understood. This study aimed to characterize RAS pathway activity subtypes and develop a robust prognostic model for LUAD patients.

**Methods:**

Transcriptomic data from 624 LUAD patients (GEO datasets: GSE31210 and GSE72094) were analyzed as the training cohort, with TCGA-LUAD as validation. Consensus clustering stratified patients based on 238 RAS pathway-related genes. Candidate genes were identified through differential expression analysis and WGCNA. Machine learning algorithms (LASSO, Random Forest, SHAP) were applied to construct a prognostic risk model. Comprehensive analyses including GSEA, CIBERSORTx-based immune infiltration, ESTIMATE scoring, and drug sensitivity prediction were performed.

**Results:**

The study identified two distinct RAS pathway activity subtypes among LUAD patients. A three-gene prognostic signature (MAPK10, PLA2G12B, SHC3) was established, with the risk score serving as an independent prognostic indicator. Risk score was an independent prognostic factor. Immune landscape analysis demonstrated that high- and low-risk patients showed different expression levels of immune cells. All signature genes correlated positively with resting mast cells, while MAPK10 and PLA2G12B negatively correlated with activated CD4 + memory T cells. GSEA revealed high-risk tumors enriched in DNA replication, cell cycle, and base excision repair, whereas low-risk tumors favored drug and retinol metabolism pathways. Low-risk patients exhibited lower TIDE and exclusion scores, indicating better immunotherapy response potential. Eight therapeutic compounds (genistein, metformin, quercetin, JNK-9 L) demonstrated favorable binding to signature genes.

**Conclusion:**

This study established a comprehensive landscape of RAS pathway activity subtypes in LUAD, identifying MAPK10, PLA2G12B, and SHC3 as novel prognostic biomarkers with significant associations with immune microenvironment remodeling, providing new insights for personalized treatment strategies and therapeutic target development.

**Supplementary Information:**

The online version contains supplementary material available at 10.1007/s12672-026-05282-9.

## Introduction

Lung adenocarcinoma (LUAD) is the most common pathologic type of Non-small cell lung cancer (NSCLC), accounting for more than 40% of the total incidence of lung cancer, and is one of the leading causes of cancer-related deaths globally [1]. Despite advances in genetic testing and targeted therapies that have improved outcomes for select patient populations, the overall prognosis of LUAD remains unsatisfactory due to its pronounced molecular heterogeneity and complex pathogenesis [2]. Consequently, identifying key oncogenic pathways, establishing novel molecular classification systems, and discovering prognostic biomarkers are critical for advancing precision medicine in lung cancer management.

The RAS signaling pathway serves as a central regulator of cell proliferation, differentiation, and survival, with dysregulation implicated in numerous malignancies [3]. KRAS mutations result in the production of a mutant KRAS protein with a reduced conversion rate from KRAS-GTP to KRAS-GDP, resulting in a constitutively active KRAS that can activate intracellular signaling cascades independently of extracellular signals, leading to tumor cell proliferation, survival, and differentiation [4]. LUAD remains the most prevalent histological subtype of NSCLC and a leading cause of cancer-related mortality worldwide [5]. In LUAD, mutations in RAS gene family members—particularly KRAS, followed by NRAS and HRAS—are exceptionally prevalent, with KRAS mutations occurring in approximately 7% of all cancer mortality and representing one of the most common oncogenic drivers [6, 7]. Despite the recent approval of KRAS inhibitors targeting KRASG12C, clinical efficacy remains modest with overall response rates (ORRs) below 50% and limited durability of response [8]. Importantly, the degree of activation of the RAS pathway in LUAD patients is not determined by mutation status alone, but is influenced by a combination of upstream and downstream regulatory mechanisms [9]. This complexity renders mutation-based assessment insufficient for comprehensively characterizing pathway activity. Systematically profiling RAS pathway expression patterns and their heterogeneity in LUAD patients holds substantial promise for refining patient stratification, risk assessment, and individualized therapeutic strategy development.

The immune microenvironment of lung tissue consists of a variety of immune cells and cytokines that play an important role in maintaining local immune homeostasis and anti-tumor immunity [10]. Recent evidence further demonstrates that metabolic reprogramming signatures, such as those centered on SLC25A1, can serve as robust biomarkers for immunotherapy response prediction in LUAD [11], underscoring the interconnection between metabolic and immune axes in this disease. Emerging evidence indicates that RAS pathway activation profoundly reshapes the tumor immune landscape by modulating immune cell infiltration, cytokine production, and immune checkpoint expression [12]. Specifically, oncogenic KRAS has been shown to promote an immunosuppressive microenvironment through upregulation of PD-L1, recruitment of myeloid-derived suppressor cells, and exclusion of cytotoxic T lymphocytes [13]. The inherent heterogeneity of LUAD leads to substantial alterations in the immune microenvironment, which not only influences disease progression but also critically impacts treatment response and patient prognosis [14]. Understanding the interplay between RAS pathway activity and immune microenvironment characteristics is therefore essential for predicting immunotherapy efficacy and designing rational combination treatment strategies.

The advent of high-throughput sequencing technology has revolutionized tumor molecular characterization, with Bulk RNA-seq emerging as a powerful tool for comprehensive transcriptomic profiling [15]. Through genome-wide expression analysis, Bulk RNA-seq provides insights into driver genes, critical pathways, and molecular subtypes [16], establishing a robust foundation for investigating RAS pathway activity in LUAD [17].

Consensus Clustering (CC), an unsupervised machine learning method, has gained widespread application in tumor molecular classification due to its superior robustness in handling high-dimensional, biologically heterogeneous datasets [18–20]. Meanwhile, interpretable machine learning (IML) has gained prominence in biomedical research [21], facilitating identification of key genes and construction of risk models by elucidating feature contributions to model outputs [22]. Given the substantial inter-patient heterogeneity in LUAD prognosis, integrating RAS pathway activity subtypes with RNA-seq data and machine learning algorithms can construct multidimensional risk models to predict survival outcomes and treatment responses, supporting personalized treatment planning [23–25].

Unlike previous studies that primarily focused on single-gene or mutation-based RAS pathway assessment, the present study innovates by integrating consensus clustering of 238 RAS pathway genes to define activity subtypes, combining interpretable machine learning (LASSO, Random Forest, SHAP) with a multi-dimensional immune landscape analysis, and further validating findings at single-cell resolution. This multilevel integrative framework moves beyond conventional prognostic modeling to provide a comprehensive molecular portrait of RAS pathway heterogeneity in LUAD. Based on GEO database and TCGA-LUAD cohort, candidate prognostic genes were prioritized through differential expression analysis, Weighted Gene Co-expression Network Analysis (WGCNA), and protein-protein interaction (PPI) network construction. A multigene prognostic risk score model was subsequently developed using LASSO regression and the SHAP interpretable algorithm, with validation in an independent cohort. We further characterized immune microenvironment differences between risk groups and predicted immunotherapy response using ESTIMATE scoring and TIDE modeling. Finally, drug prediction analysis coupled with molecular docking identified potential therapeutic compounds targeting signature genes. This integrative approach combining transcriptomic profiling, consensus clustering, and machine learning offers novel insights for LUAD molecular classification and precision oncology.

## Methods

### Data acquisition and preprocessing

LUAD transcriptomics datasets GSE31210 and GSE72094 were retrieved from the Gene Expression Omnibus (GEO) database. GSE31210, profiled on GPL570 platform ([HG-U133_Plus_2] Affymetrix Human Genome U133 Plus 2.0 Array), comprised 226 tumor and 20 normal lung specimens. GSE72094, generated using GPL15048 platform (Rosetta/Merck Human RSTA Custom Affymetrix 2.0 microarray), contained 442 LUAD samples. TCGA-LUAD transcriptomic profiles, along with corresponding clinical variables (age, sex, tumor stage) and survival outcomes, were downloaded from the GDC portal. The supplementary validation dataset GSE13213, profiled on GPL570 platform, includes 117 LUAD samples.

The single-cell RNA sequencing (scRNA-seq) data was downloaded from the Code Ocean platform (https://codeocean.com/capsule/8321305/tree/v1). This dataset contains single-cell transcriptome data from 10 LUAD tumor tissues and 10 control lung tissues, providing an important resource for analyzing the cellular composition and gene expression heterogeneity of the lung adenocarcinoma tumor microenvironment. For single-cell RNA sequencing analysis, data were obtained from a publicly available dataset of human LUAD (The article directly provides the data download link - Single-cell RNA sequencing of human lung adenocarcinomas - 10.24433/CO.0121060.v1).

A total of 238 RAS pathway genes were obtained from the KEGG database.

### Consensus clustering analysis based on RAS pathway–related genes

Consensus Clustering was performed on 624 LUAD samples using 238 RAS pathway genes via “ConsensusClusterPlus” R package with 1,000 iterations. Clustering robustness was assessed, and t-SNE analysis visualized sample distribution.

### Differential expression analysis of transcriptomic data

The “limma” R package identified DEGs between subgroups using criteria of |log_2_FC| > 0.5 and *p* < 0.05. Results were visualized through volcano plots (“ggplot2”) and heatmaps (“pheatmap”).

### Weighted gene co-expression network analysis (WGCNA)

WGCNA identified co-expression modules and hub genes in the training cohort. The pickSoftThreshold function determined optimal soft-thresholding power (β) for scale-free topology. After constructing the adjacency matrix and Topological Overlap Matrix (TOM), hierarchical clustering with dynamic tree cut identified gene modules (minimum size: 30, merge height: 0.25). Module eigengenes represented each module’s expression pattern. Pearson correlation evaluated associations between module eigengenes (MEs) and LUAD subtypes, selecting modules with *P* < 0.05. Hub genes were defined by GS > 0.3. Final candidates were identified by intersecting DEGs, RAS pathway genes, and WGCNA hub genes.

### Enrichment analysis and protein–protein interaction (PPI) network construction

Gene Ontology (GO) and KEGG enrichment analyses were conducted using “clusterProfiler” R package (*p* < 0.05). PPI networks were constructed via STRING database (species: Homo sapiens; minimum score: 0.4) and visualized in Cytoscape.

### Development and validation of a prognostic risk scoring model

Univariate Cox regression identified prognosis-associated candidates. LASSO regression (“glmnet” package with 10-fold cross-validation) and Random Forest (“randomForest” package evaluating Gini importance) were applied. Genes selected by both algorithms were designated prognostic markers.

Risk scores were calculated as: $\:risk\:score=\sum_{j=1}^{n}\left({Coef}_{j}\times\:{X}_{j}\right)$. Patients were stratified into high- and low-risk groups using optimal cutoff values. “survminer” R package generated Kaplan–Meier (K-M) survival curves (log-rank test, *p* < 0.05). The “ggrisk” package visualized risk score distribution, survival status, and prognostic gene expression heatmaps. “timeROC” R package produced Receiver Operating Characteristic (ROC) curves and calculated Area Under the Curve (AUC). Identical analyses on TCGA dataset validated model consistency.

### Independent prognostic analysis and construction of a nomogram

Univariate Cox regression screened potential prognostic factors (*p* < 0.05, HR ≠ 1) among risk scores and clinical features, followed by proportional hazards testing (*p* > 0.05). Variables meeting PH assumptions underwent multivariate analysis to identify independent predictors. A nomogram predicting 1-, 3-, and 5-year survival was constructed (“rms” package) with calibration curves (“regplot”), ROC curves (“timeROC”), and decision curve analysis (“ggDCA”).

### Clinical feature correlation analysis

Wilcoxon rank-sum tests compared risk scores and prognostic gene expression across clinical subgroups (age, sex, stage) in the training cohort (*p* < 0.05).

### Gene set enrichment analysis (GSEA)

Differential expression analysis (“limma”) generated ranked gene lists by log_2_FC. GSEA was performed using “clusterProfiler” with KEGG gene sets from MSigDB.

### Immune infiltration analysis and ESTIMATE analysis

CIBERSORTx quantified 22 immune cell types in training samples. Wilcoxon tests compared immune infiltration between risk groups. Pearson correlation assessed relationships among immune cells and between key genes and immune populations.

ESTIMATE algorithm calculated immune, stromal, and ESTIMATE scores from expression matrices. Wilcoxon tests compared scores between groups. Immune checkpoint gene expression and TIDE scores (immune dysfunction, exclusion) were similarly evaluated.

### Drug sensitivity prediction and molecular docking

The Connectivity Map (CMap) database screened therapeutic compounds by uploading candidate gene lists for enrichment analysis. Molecules with negative connectivity scores, indicating potential reversal of LUAD expression signatures, were prioritized and validated through molecular docking for binding affinity assessment.

### Single-cell RNA sequencing data analysis and validation of key regulatory gene expression

A systematic pipeline was applied for in-depth analysis of single-cell RNA sequencing data. Data preprocessing and quality control were performed using the Seurat package (version 5.1.0). Cells were retained based on standard quality control criteria, requiring a detected gene count between 200 and 8,000 per cell and a mitochondrial gene expression proportion of less than 15%. Gene expression was subsequently normalized using the LogNormalize method, and the 2,000 most highly variable genes were identified via the FindVariableFeatures function to serve as the feature gene set for downstream analyses.

For data integration and batch effect correction, principal component analysis (PCA) was first applied to reduce the dimensionality of the expression matrix. The Harmony algorithm was then employed to integrate and correct the principal component space based on covariates such as sample origin, effectively removing technical batch effects while preserving genuine biological variation to the greatest extent possible.

Cell clustering and visualization were subsequently performed on the batch-corrected data. Gene expression values of the selected feature genes were centered and scaled using the ScaleData function. A shared nearest neighbor (SNN) graph was constructed based on the corrected principal components, and cell clusters were identified using the Louvain algorithm. Uniform Manifold Approximation and Projection (UMAP) was applied for nonlinear dimensionality reduction and visualization, enabling intuitive representation of cell distribution in low-dimensional space and inter-group differences. Finally, the expression of key prognostic genes was validated at single-cell resolution.

## Results

### Identification of RAS subtypes in LUAD patients

**Fig. 1:**
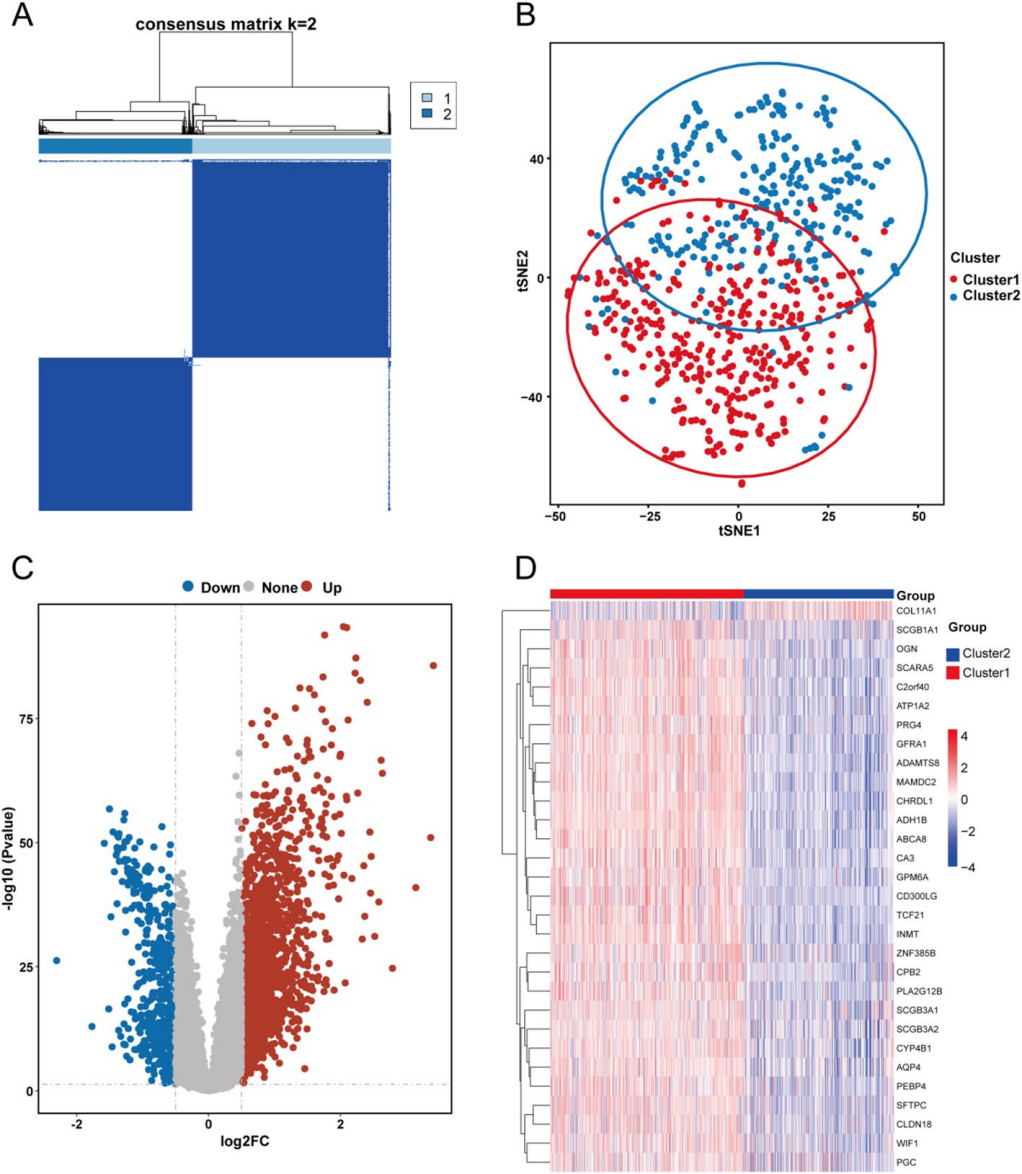
Unsupervised clustering results and differential expression gene selection. **A** Unsupervised clustering results plot. **B** t-SNE plot of clustering groups, with red dots representing Cluster 1 samples and blue dots representing Cluster 2 samples. **C** Volcano plot of differential analysis, with red dots representing upregulated genes, blue dots representing downregulated genes, and gray dots representing non-differential genes. **D** Heatmap showing the expression patterns of the top 30 significantly upregulated and downregulated DEGs in Cluster 1 and Cluster 2 samples

We extracted 624 LUAD patient samples with clinical information from GEO datasets. Consensus clustering analysis using 238 RAS gene expression profiles stratified patients into two distinct subgroups: Cluster 1 and Cluster 2 (Fig. 1A). t-SNE analysis confirmed substantial differences in RAS gene expression patterns between subgroups (Fig. 1B). Between the two subgroups, 2257 DEGs were detected, comprising 1736 upregulated and 521 downregulated genes (Fig. 1C). The top 30 DEGs are displayed in a heatmap (Fig. 1D).

### WGCNA

**Fig. 2:**
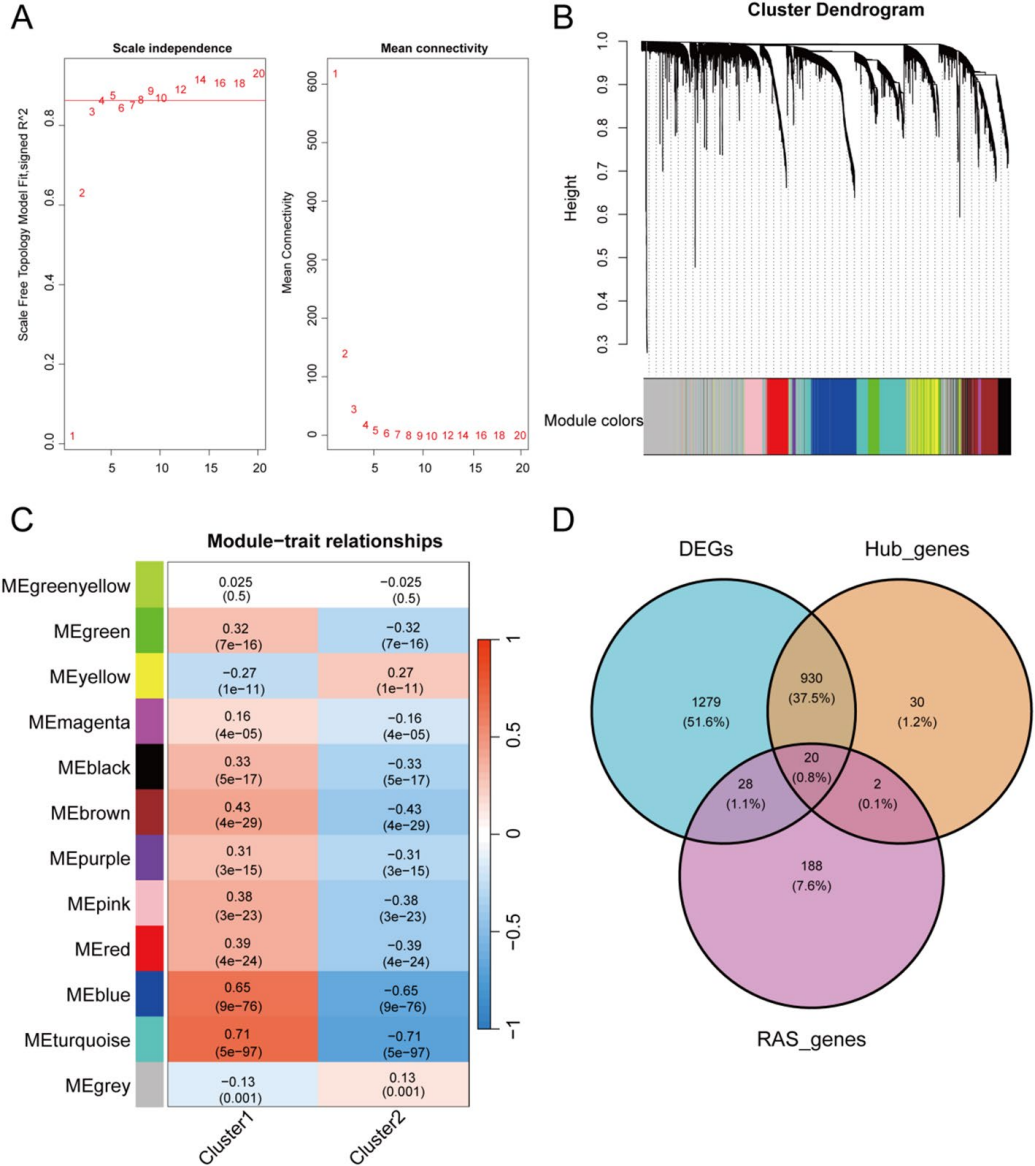
WGCNA analysis. **A** Plot of the independence of the dataset and soft thresholding values, along with connectivity. **B** Dendrogram of hierarchical clustering. **C** Heatmap of the correlation analysis between module feature genes and subtype grouping. **D** Venn diagram of the identified candidate genes

We performed WGCNA using gene expression data to detect LUAD-associated module genes. The pickSoftThreshold function tested various soft-thresholding values (β) to construct the adjacency matrix with optimal parameters (Fig. 2A). This matrix was converted into a Topological Overlap Matrix (TOM) representing gene co-expression similarity. Applying hierarchical clustering with dynamic tree-cutting (minimum module size: 30, maximum distance: 0.25) identified 12 gene modules (Fig. 2B). We then assessed correlations between modules and consensus clustering subtypes (Cluster 1 vs. Cluster 2). The grey module was excluded to minimize analytical noise. Two modules (blue and turquoise) demonstrated correlation coefficients exceeding 0.5 with subtype features (Fig. 2C), suggesting strong associations with LUAD progression. These key modules yielded 982 hub genes. Integrating DEGs, module hub genes, and RAS genes identified 20 overlapping candidate genes (Fig. 2D) considered highly relevant to disease pathogenesis.

### Enrichment analysis and PPI of candidate genes

**Fig. 3:**
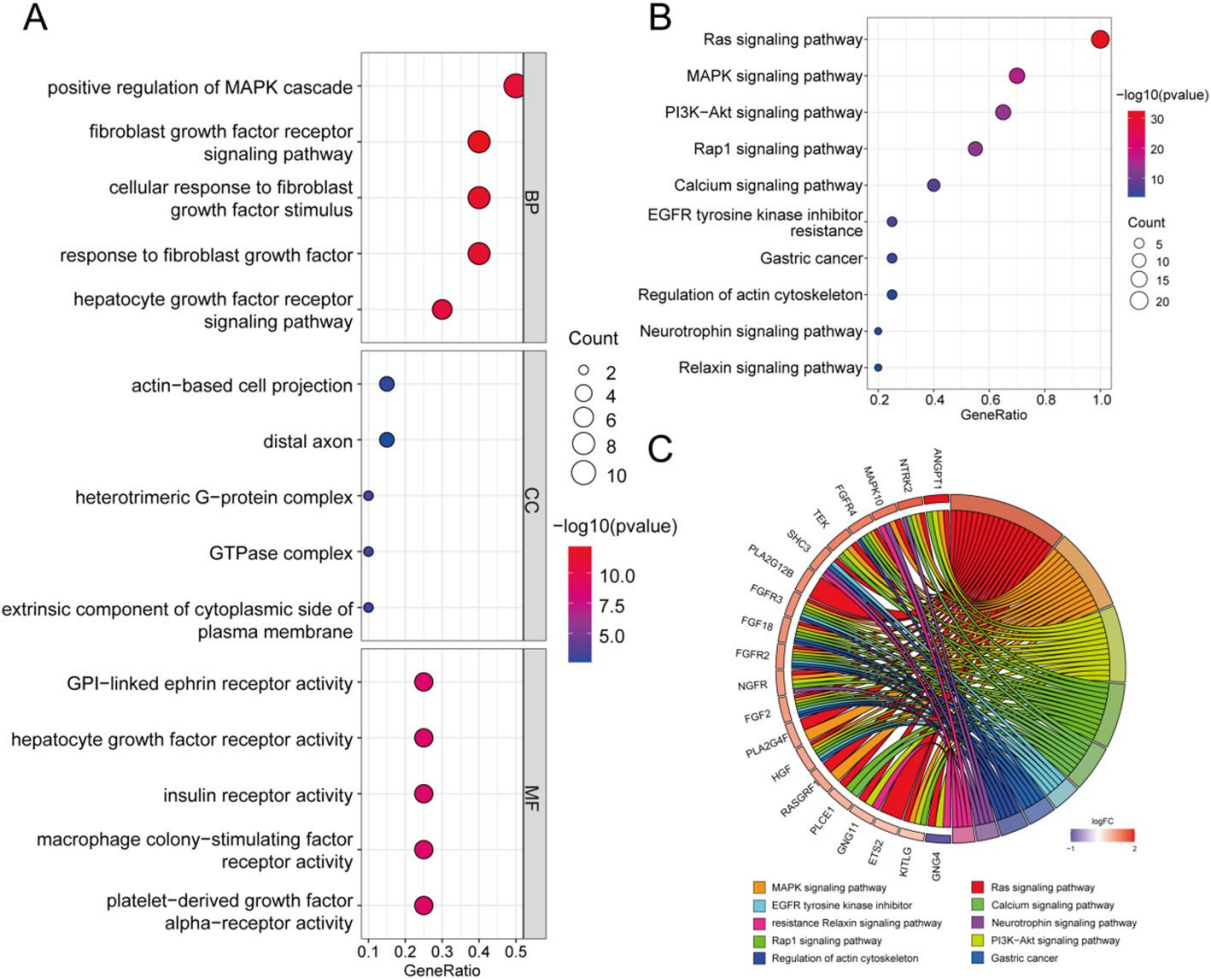
GO and KEGG enrichment analysis. **A** Top 5 pathways in GO enrichment for CC, BP, and MF. **B** Top 10 pathways in KEGG enrichment. **C** Gene association plot for the top 10 pathways in KEGG enrichment analysis

GO and KEGG functional enrichment analyses were performed on the 20 selected candidate genes. In the GO enrichment analysis, these genes were primarily enriched in biological processes (BP) such as positive regulation of MAPK cascade, fibroblast growth factor receptor signaling pathway, cellular response to fibroblast growth factor stimulus, response to fibroblast growth factor, hepatocyte growth factor receptor signaling pathway; cellular components (CC) such as actin-based cell projection, distal axon, heterotrimeric G-protein complex, GTPase complex, extrinsic component of cytoplasmic side of plasma membrane; and molecular functions (MF) such as GPI-linked ephrin receptor activity, hepatocyte growth factor receptor activity, insulin receptor activity, macrophage colony-stimulating factor receptor activity, platelet-derived growth factor alpha-receptor activity (Fig. 3A). KEGG enrichment analysis revealed that these genes are involved in signaling pathways such as Ras signaling pathway, MAPK signaling pathway, PI3K-Akt signaling pathway, Rap1 signaling pathway, Calcium signaling pathway (Fig. 3B-C).

PPI network construction for the 20 genes explored regulatory mechanisms in disease progression, yielding a network with 12 genes and 53 interactions. Topological analysis revealed that NGFR, FGFR2, NTRK2, and FGF18 possessed higher central connectivity (Degree values), suggesting their pivotal regulatory roles through interactions with other genes (Supplementary Fig. 1).

### Identification of prognostic genes and explainable machine learning algorithms

**Fig. 4:**
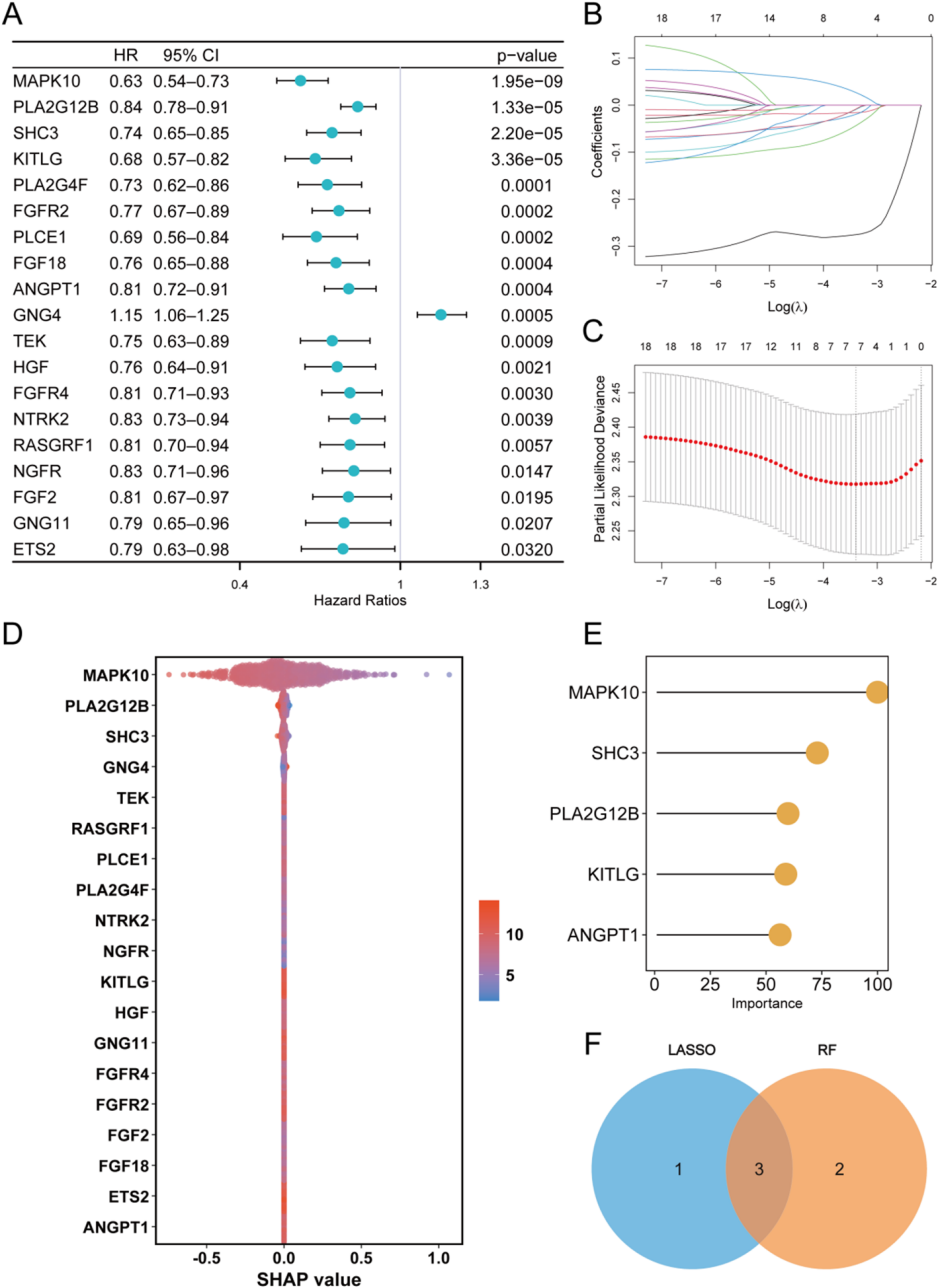
Prognostic gene selection. **A** Univariate Cox regression analysis of candidate genes. **B** LASSO regression coefficient path plot. **C** LASSO regression cross-validation curve. **D** SHAP model explaining the importance of prognostic genes in the model. **E** Importance ranking of key genes selected by the RF algorithm. **F** Venn diagram of prognostic genes selected by both RF and LASSO algorithms

Univariate Cox regression analysis using “survival” R package screened candidate genes in the training set, identifying 19 potential prognostic genes (Fig. 4A). Following proportional hazards assumption testing (PH > 0.05), qualified genes underwent LASSO regression via “glmnet” R package with 10-fold cross-validation (Fig. 4B and C). SHAP plots illustrated feature gene importance in LUAD prognosis prediction (Fig. 4D), showing MAPK10, PLA2G12B, and SHC3 as primary negative contributors and GNG4 as the main positive contributor. RF algorithm identified five feature genes (Fig. 4E). Intersection of both algorithm results yielded three key genes: MAPK10, PLA2G12B, and SHC3 (Fig. 4F).

### Construction and validation of prognostic risk model

**Fig. 5:**
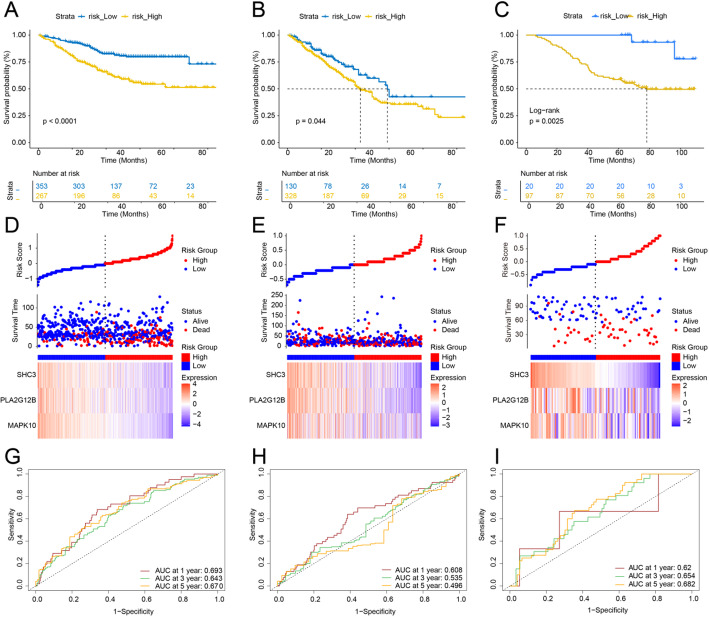
Prognostic performance of the three-gene risk score model in the training cohort, validation cohort, and external validation dataset. **A**–**C** Kaplan-Meier survival curves comparing overall survival between high- and low-risk groups in the training cohort (**A**), validation cohort (**B**), and external validation dataset GSE13213 (**C**). The number of patients at risk at each time point is shown below the curves. **D**–**F** Risk score distribution, survival status, and expression heatmap of the three model genes (SHC3, PLA2G12B, and MAPK10) in the training cohort (**D**), validation cohort (**E**), and GSE13213 dataset (**F**). **G**–**I** Time-dependent ROC curves assessing the predictive accuracy of the risk model for 1-, 3-, and 5-year survival in the training cohort (**G**), validation cohort (**H**), and GSE13213 dataset (**I**).

Risk score calculation stratified all LUAD patients into high- and low-risk groups. Kaplan-Meier analysis demonstrated that low-risk patients had significantly prolonged OS compared to high-risk patients in the training cohort (*p* < 0.0001, Fig. 5A). Risk score distribution, survival status, and expression heatmaps of SHC3, PLA2G12B, and MAPK10 were visualized using the “ggrisk” package (Fig. 5D), revealing that higher risk scores were associated with increased mortality and distinct gene expression patterns. Time-dependent ROC analysis in the training cohort yielded 1-year, 3-year, and 5-year AUC values of 0.693, 0.643, and 0.670, respectively (Fig. 5G). The model was subsequently validated in the TCGA validation cohort, which confirmed significantly better prognosis in low-risk patients (*p* = 0.044, Fig. 5B), with consistent expression-outcome associations observed in the risk heatmap (Fig. 5E) and 1-year, 3-year, and 5-year AUC values of 0.608, 0.535, and 0.496 (Fig. 5H). Further external validation in the GSE13213 dataset demonstrated a significant survival difference between risk groups (*p* = 0.0025, Fig. 5C), with risk score distribution and gene expression patterns consistent with the training cohort (Fig. 5F), and notably improved long-term predictive performance with 1-year, 3-year, and 5-year AUC values of 0.620, 0.654, and 0.682, respectively (Fig. 5I). Collectively, these results support the robustness and generalizability of the three-gene prognostic model across independent cohorts.

### Independent prognostic analysis and nomogram construction

**Fig. 6:**
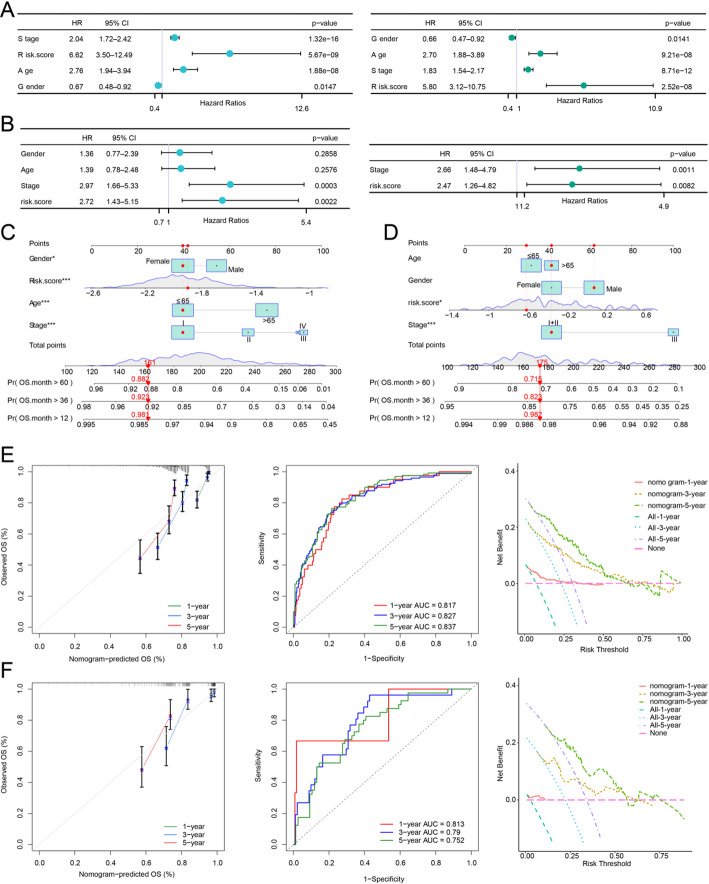
Nomogram construction and external validation. **A** Univariate and multivariate Cox regression analyses in the training cohort. **B** Univariate and multivariate Cox regression analyses in the external validation dataset GSE13213. **C** Nomogram integrating risk score and clinical features for individualized OS prediction in the training cohort. **D** Nomogram construction and evaluation in the GSE13213 dataset. **E** Calibration curves, time-dependent ROC curves, and decision curve analysis of the nomogram in the training cohort. **F** Calibration curves, time-dependent ROC curves, and decision curve analysis of the nomogram in the GSE13213 dataset

To assess the independent prognostic value of the risk score, univariate and multivariate Cox regression analyses were performed incorporating gender, age, stage, and risk score. In the training cohort, univariate analysis identified risk score, age, stage, and gender as significant prognostic factors, and multivariate analysis confirmed risk score as an independent predictor of OS (Fig. 6A). These findings were further corroborated in the external validation dataset GSE13213, where risk score retained its independent prognostic significance upon multivariate adjustment (Fig. 6B).

A nomogram integrating risk score with clinical variables was subsequently constructed to enable individualized prediction of 1-year, 3-year, and 5-year OS in the training cohort (Fig. 6C), and was independently evaluated in the GSE13213 dataset (Fig. 6D). In the training cohort, calibration curves demonstrated good agreement between nomogram-predicted and observed survival probabilities, time-dependent ROC analysis yielded 1-year, 3-year, and 5-year AUC values of 0.817, 0.827, and 0.837, respectively, and DCA confirmed positive net clinical benefit across a wide range of risk thresholds (Fig. 6E). External validation in GSE13213 similarly showed satisfactory calibration, with 1-year, 3-year, and 5-year AUC values of 0.813, 0.790, and 0.752, and favorable DCA results (Fig. 6F), collectively supporting the robustness and clinical applicability of the nomogram.

### scRNA-seq analysis for exploring cellular heterogeneity

**Fig. 7:**
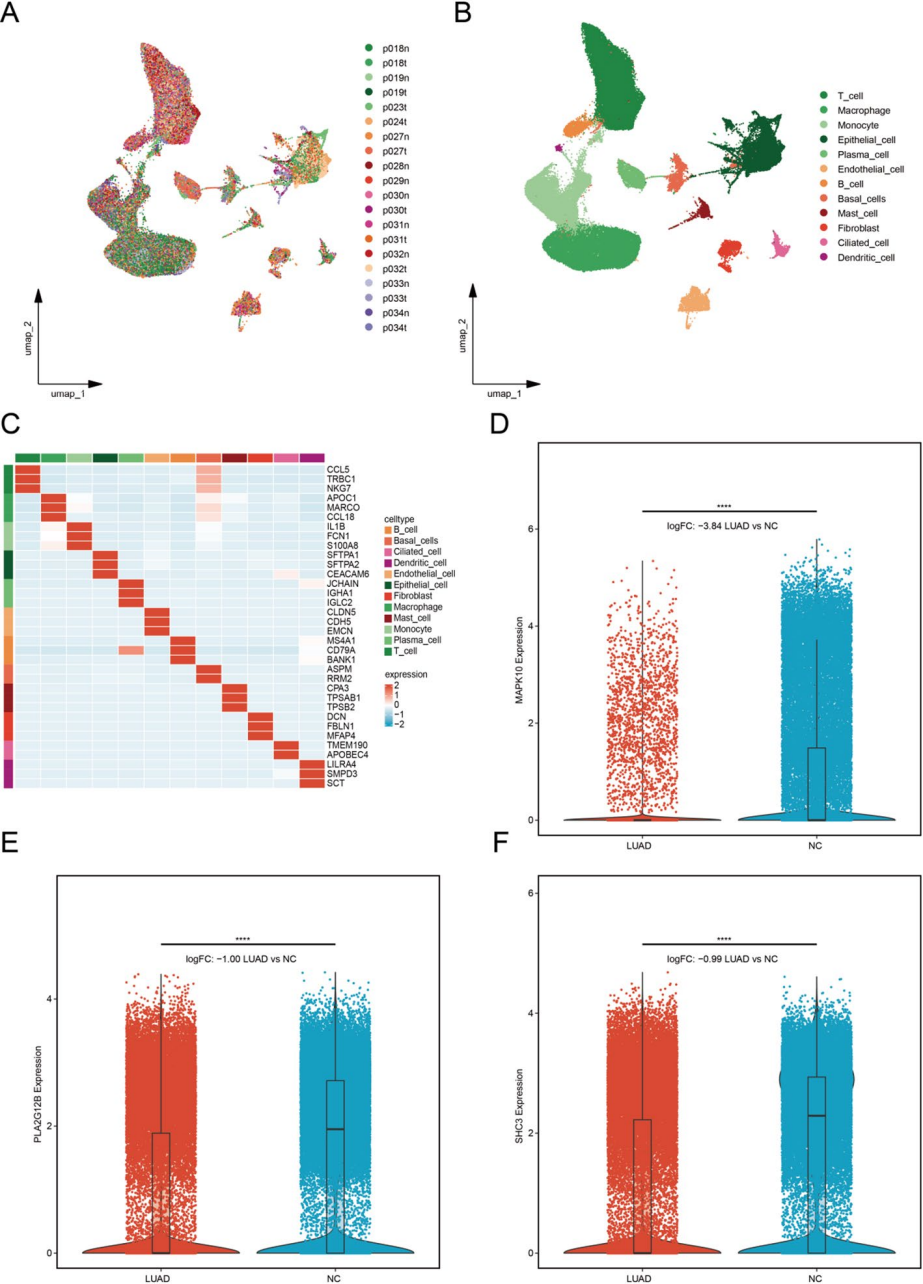
Cellular heterogeneity and Key Gene Validation in LUAD Using Single-Cell RNA Sequencing. **A** UMAP plot following Harmony-based batch correction, with each sample colored distinctly to demonstrate effective removal of batch effects. **B** UMAP plot of cell type distribution annotated by canonical marker genes, with 12 distinct cell types identified and represented by different colors. **C** Heatmap of marker genes for each cell type, where color intensity reflects the average gene expression level. **D**–**F** Differential expression analysis of MAPK10 (**D**), PLA2G12B (**E**), and SHC3 (**F**) between LUAD and normal control (NC) groups at single-cell resolution.

To further explore the cellular heterogeneity of the tumor microenvironment and validate the expression of the three prognostic genes at single-cell resolution, we performed scRNA-seq analysis on LUAD samples. After Harmony-based batch correction, UMAP dimensionality reduction demonstrated effective elimination of batch effects across samples (Fig. 7A). Based on canonical marker gene expression, a total of 12 distinct cell types were identified, including T cells, macrophages, monocytes, epithelial cells, plasma cells, endothelial cells, B cells, basal cells, mast cells, fibroblasts, ciliated cells, and dendritic cells (Fig. 7B). Cell type-specific marker genes were further confirmed by heatmap visualization, in which each cell population exhibited characteristic expression patterns consistent with their assigned identities (Fig. 7C).

We next examined the expression of the three key genes across LUAD and adjacent normal control (NC) cells. MAPK10 expression was significantly downregulated in LUAD compared to NC ( Fig. 7D), as were PLA2G12B (Fig. 7E) and SHC3 (Fig. 7F). These findings at single-cell resolution are consistent with bulk RNA-seq results, reinforcing the biological relevance of the three-gene signature and suggesting that their downregulation may reflect tumor-associated transcriptional alterations within the LUAD microenvironment.

### Correlation analysis of clinical features

**Fig. 8:**
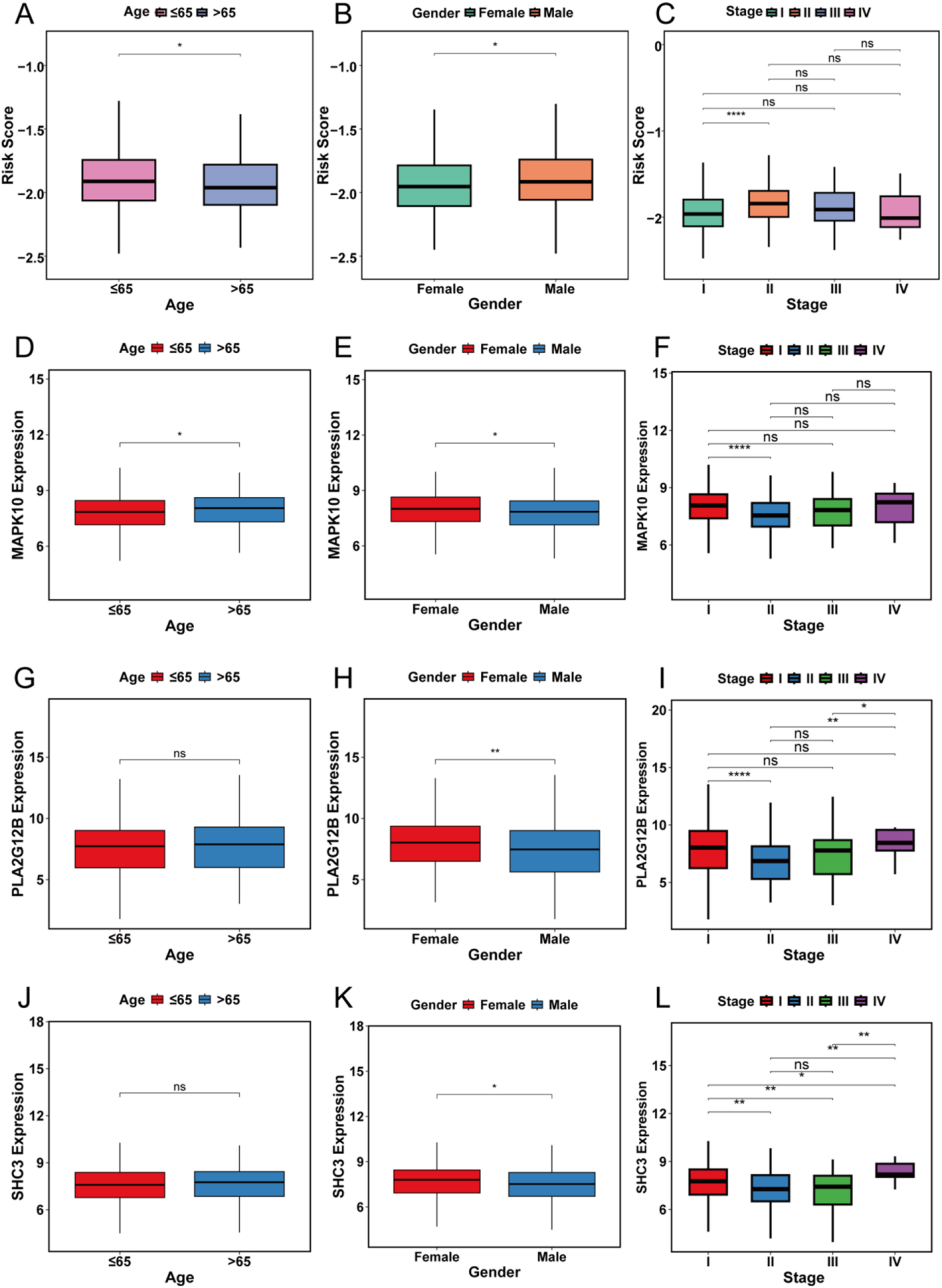
Differences in Prognostic Gene Expression and Risk Scores Across Clinical Feature Subgroups. **A**–**C** Comparison of differences in risk scores across age, gender, and stage subgroups. **D**–**L** Comparison of differences in prognostic gene expression across age, gender, and stage subgroups. ns represents for no significant difference, *P < 0.05, **P < 0.01, ***P < 0.001, ****P < 0.0001

Risk score correlations with clinical features revealed significant differences across subgroups, with elevated scores in patients < 65 years, male patients, and stage II cases (Fig. 8A**–**C).

Prognostic gene expression analysis across clinical subgroups showed: MAPK10 exhibited significant variation across multiple subgroups (Fig. 8D–F); PLA2G12B and SHC3 displayed differential expression across gender and stage categories (Fig. 8G–L). These patterns suggest important roles in tumor progression and prognosis.

### GSEA

**Fig. 9:**
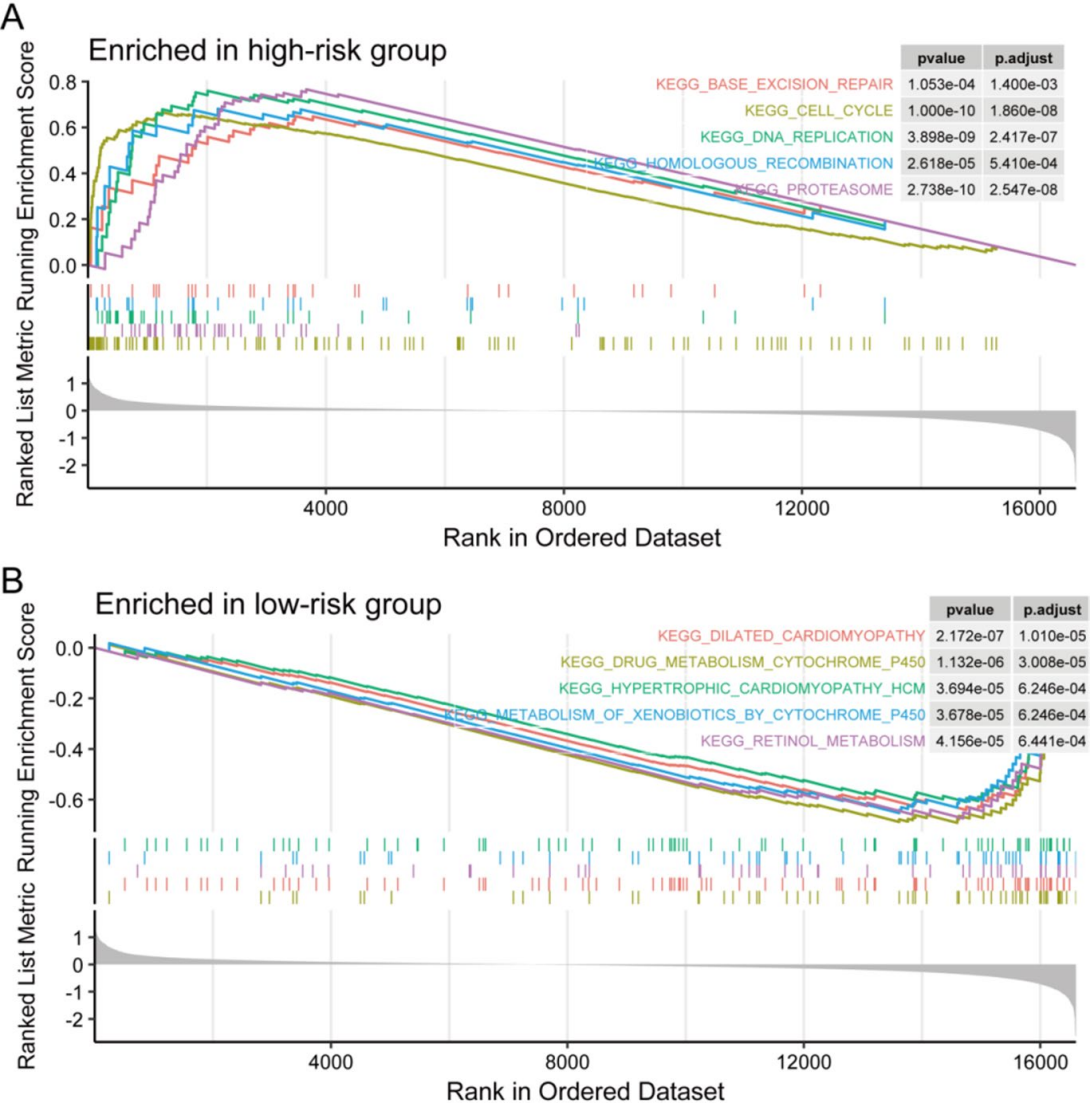
GSEA Enrichment Analysis. **A** Top five GSEA gene sets in high-risk LUAD. **B** Top five GSEA gene sets in low-risk LUAD

GSEA showed that the differential genes in the high-risk group were primarily enriched in pathways such as Base excision repair, Cell cycle, DNA replication, Homologous recombination, and Proteasome (Fig. 9A). The differential genes in the low-risk group were mainly associated with pathways related to Dilated cardiomyopathy, Drug metabolism cytochrome P450, Hypertrophic cardiomyopathy hcm, Metabolism of xenobiotics by cytochrome P450, and Retinol metabolism (Fig. 9B).

### Immune infiltration analysis and ESTIMATE analysis

We applied CIBERSORT algorithm to training dataset gene expression profiles, estimating immune cell subpopulation abundances (T cells, B cells, natural killer cells, macrophages) in LUAD tissues.

Stacked bar charts displayed 22 immune cell types across risk groups (Supplementary Fig. 2A). High-risk groups showed significant upregulation of plasma cells, CD8 + T cells, activated CD4 + memory T cells, resting NK cells, M0 macrophages, M1 macrophages, and neutrophils (*P* < 0.05), while memory B cells, resting CD4 + memory T cells, gamma delta T cells, monocytes, resting dendritic cells, resting mast cells, and eosinophils were downregulated (*P* < 0.05) (Supplementary Fig. 2B). Correlation analysis identified strongest positive correlation between activated CD4 + memory T cells and CD8 + T cells, and strongest negative correlation between CD8 + T cells and resting CD4 + memory T cells (Supplementary Fig. 2C). All three prognostic genes correlated positively with resting mast cells, while MAPK10 and PLA2G12B showed negative correlations with activated CD4 + memory T cells (Supplementary Fig. 2D). These findings indicate potential regulatory roles of prognostic genes in immune modulation and microenvironment dynamics.

**Fig. 10:**
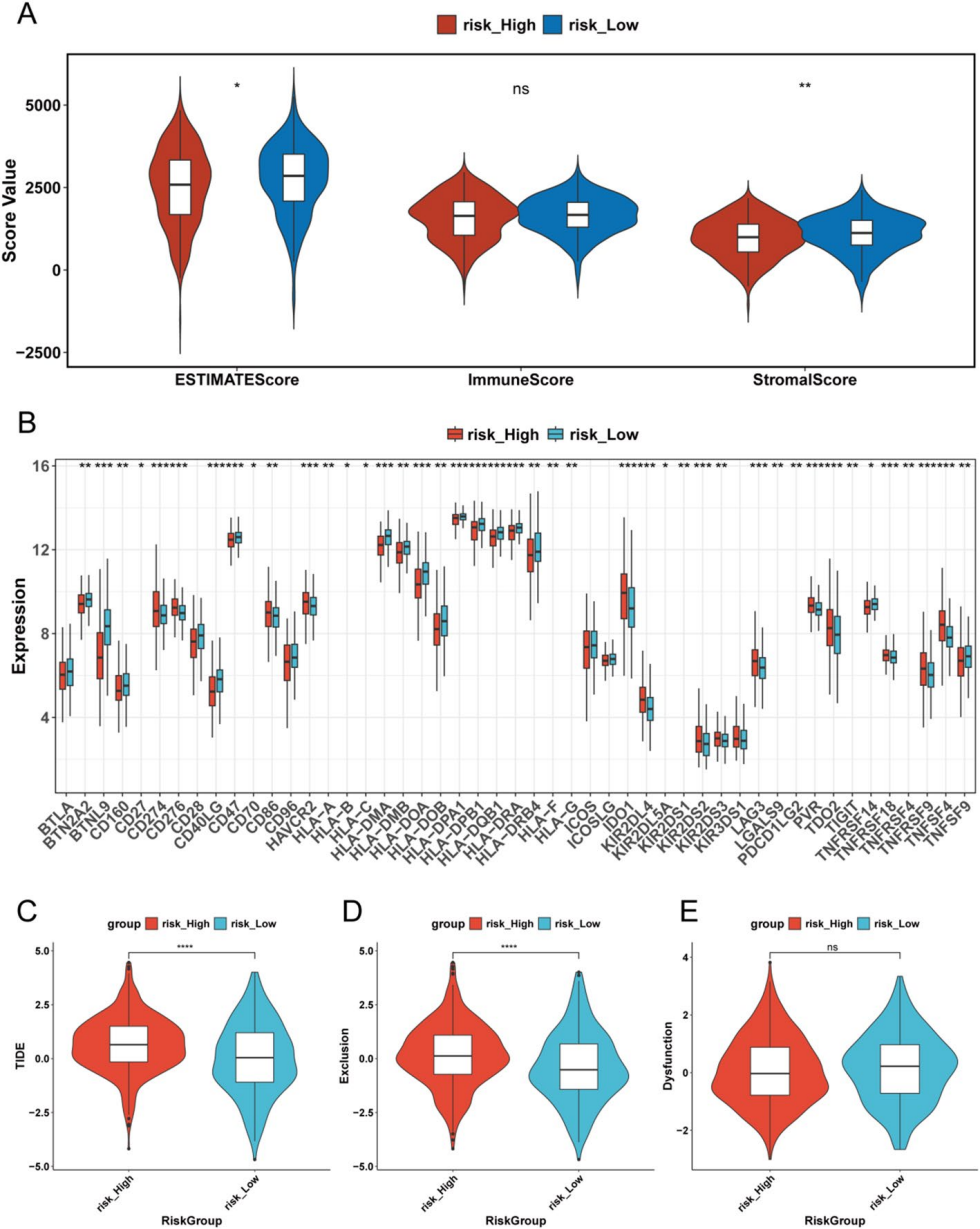
Analysis of immunotherapy. **A** Stromal, immune, and ESTIMATE score comparisons. **B** Differential immune checkpoint gene expression. **C**–**E** TIDE, exclusion, and dysfunction score comparisons. ns, no significant difference; *P < 0.05, **P < 0.01, ***P < 0.001, ****P < 0.0001

ESTIMATE algorithm calculated stromal, immune, and ESTIMATE scores for each patient. Significant differences in stromal and ESTIMATE scores emerged between risk groups, while immune scores showed no difference (Fig. 10A). Immune checkpoint gene comparison revealed elevated BTN2A2, BTNL9, and CD47 expression in low-risk LUAD (Fig. 10B). Low-risk subgroups exhibited significantly reduced TIDE and Exclusion scores, suggesting enhanced immunotherapy responsiveness (*p* < 0.05) (Fig. 10C–E).

### Drug sensitivity prediction and molecular docking

**Fig. 11:**
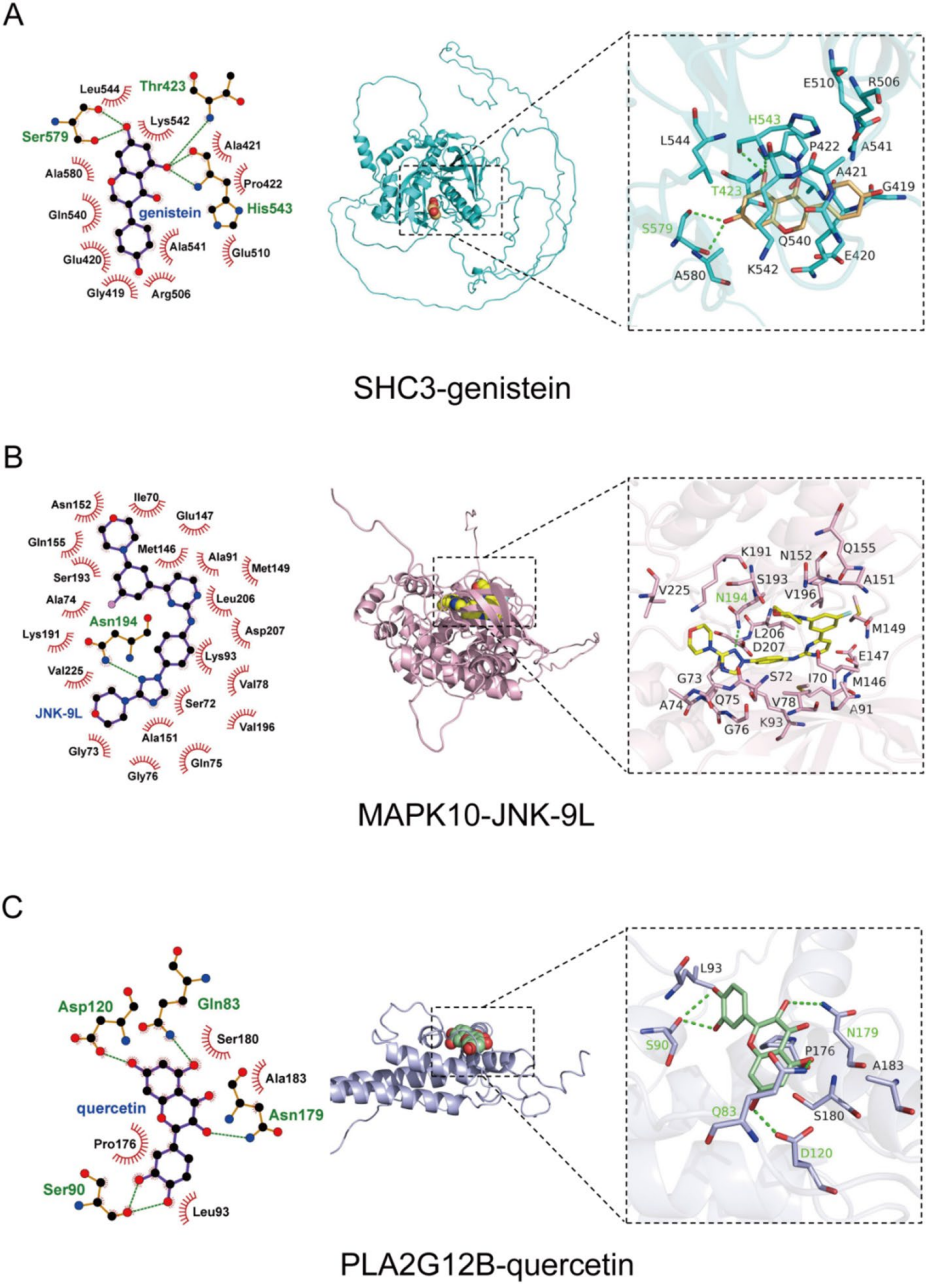
Interaction diagrams of small molecules with target proteins. **A** SHC3 with genistein. **B** MAPK10 with JNK-9 L. **C** PLA2G12B with quercetin.

Eight potential therapeutic compounds were identified from the CMap database, including genistein, metronidazole, menadione, TG-101,348, metformin, JNK-9 L, HG-6-64-01, and quercetin (Supplementary Table 1). Molecular docking was performed, and for each key target, the ligand-receptor complex with the lowest binding energy was selected for visualization. The binding energies were − 7.486, -10.384, and − 7.091 kcal/mol, respectively. The molecular docking results are shown in Fig. 11.

## Discussion

This study adopted a multilevel integrative analysis strategy to systematically elucidate the critical role of RAS signaling pathway activity in the development and progression of LUAD. Through consensus clustering analysis based on RAS pathway-related genes, LUAD patients were divided into two distinct RAS activity subtypes (Cluster 1 and Cluster 2), providing a novel molecular classification framework for precision medicine in LUAD. From 2257 differentially expressed genes, 20 RAS-related candidate genes were selected, with upregulated genes predominating (1736 genes), a pattern that may reflect enhanced signaling pathway activation and metabolic hyperfunction during malignant progression of LUAD cells. Through iterative screening using LASSO and Random Forest machine learning algorithms, three core diagnostic genes were ultimately identified: SHC3, MAPK10, and PLA2G12B.

The novelty of this study lies in three key aspects: (1) the systematic characterization of RAS pathway activity heterogeneity through unsupervised transcriptomic clustering rather than mutation-centric approaches; (2) the development of an interpretable three-gene prognostic signature incorporating signal transduction (SHC3, MAPK10) and lipid metabolism (PLA2G12B) dimensions; and (3) the cross-scale validation strategy spanning bulk RNA-seq, independent external cohorts, and single-cell resolution analysis.

GO and KEGG functional enrichment analyses revealed that these candidate genes were significantly enriched in RAS and its downstream MAPK, PI3K-Akt, Rap1, and calcium signaling pathways, suggesting that these genes may affect the occurrence and progression of LUAD by regulating these key pathways, which is consistent with previously reported central regulatory roles of the MAPK pathway in tumor proliferation, differentiation, and apoptosis in LUAD [26]. Notably, the synergistic activation of RAS-MAPK and PI3K-Akt pathways plays distinct but complementary roles in maintaining LUAD stem cell-like properties, providing important context for understanding the functions of the key genes in this study [27].

PPI network analysis shows that genes such as NGFR, FGFR2, NTRK2 and FGF18 have a higher connection in the network, suggesting that these genes may be at a core position in the regulatory network related to the RAS pathway and participate in regulating multiple key pathways. However, the innovation of this study is primarily concentrated on the discovery of three key genes (SHC3, MAPK10, and PLA2G12B), which participate in RAS pathway regulation through different mechanisms and possess unique clinical value. Previous studies have shown that NGFR has a dual role in a variety of tumors, and can be used as both proliferation, metastasis and may also have a tumor suppressor effect [28, 29]. FGFR2 is one of the classic receptor tyrosine kinases. It is common to increase, mutation or fusion in LUAD, resulting in enhanced activity. Activated FGFR2 can continuously stimulate the MAPK and PI3K/Akt pathways, driving tumor cell proliferation, angiogenesis and drug resistance [30]. NTRK2 mainly mediates neurotrophic factor signaling and promotes cell survival and anti-apoptotic in tumors [31]. FGF18 can activate downstream signaling pathways through binding to FGFR, promoting tumor cell motility and invasion [32]. The above results indicate that these key genes possess potential regulatory functions due to their centrality in the PPI network and warrant further in-depth investigation.

As the core findings of this study, SHC3, MAPK10, and PLA2G12B demonstrated excellent diagnostic value and prognostic prediction capability, with the combination of these three genes constituting a novel molecular marker panel. Its advantages lie in: first, they regulate RAS pathway activity at different molecular levels, forming a multi-target synergistic effect; second, compared to existing LUAD biomarkers (such as single KRAS mutation status detection), the three-gene model in this study integrates both signal transduction (SHC3 and MAPK10) and lipid metabolism (PLA2G12B) dimensions, providing a more comprehensive assessment of tumor biological characteristics. SHC3 is a member of the Src homology domain protein family, mainly involved in neurotrophic factor signaling and MAPK pathway activation. As an adaptor protein, SHC family members (including SHC1 and SHC3) play a critical role in receptor tyrosine kinase signal transduction through their unique pTB-CH1-SH2 domain structure, linking activated receptor tyrosine kinases to the Ras pathway via GRB2/SOS [33]. Notably, SHC proteins can also recruit and activate the MAPK pathway, thereby promoting cell proliferation [34]. In this study, SHC3 expression was downregulated in the high-risk group and was negatively correlated with T cell memory activation status, suggesting that it may affect the tumor immune escape process by regulating the immune microenvironment. This finding suggests that SHC3 may possess tumor suppressor functions, with its low expression leading to abnormal MAPK pathway activation while simultaneously weakening anti-tumor immune responses.

MAPK10 (also known as JNK3) belongs to the mitogen-activated protein kinase family and is an important factor in regulating the apoptosis and stress response of cells. As a member of the JNK subfamily, MAPK10 participates in multiple key physiological processes including apoptosis, differentiation, and proliferation. Stress-induced JNK activation can lead to cell death through activation of the mitochondrial apoptotic pathway in many cell types [35]. Studies have shown that low expression of MAPK10 is closely related to the adverse prognosis of LUAD patients, and may play a carcinogenic role by inhibiting cell programmed death and promoting tumor cell survival and metastasis [36].

PLA2G12B is a member of the secretory phospholipase A2 family, involved in lipid metabolism and cellular signal transduction regulation. Secretory phospholipase A2 (sPLA2), in addition to playing key roles in tumor proliferation and progression, also serves as an integrated regulator of immune and metabolic responses [37, 38]. Studies on PLA2G2A (another member of the PLA2 family) indicate that proinflammatory cytokines such as IL-1β, IFN-γ, TNF-α, and IL-6 can increase its expression in prostate cancer cell lines through various signaling mechanisms involving activation of NF-κB and STAT family transcription factors [39]. While the specific regulatory mechanisms of PLA2G12B in lung cancer require further investigation, the broader PLA2 family’s role in inflammation-driven carcinogenesis is well established. This study found that its expression was significantly correlated with changes in immune cell infiltration levels, and may participate in the LUAD progression by modulating the inflammatory response in the tumor microenvironment. Combined with the nomogram model results, these genes have high diagnostic value in risk score prediction and LUAD prognosis judgment, suggesting that they are expected to become potential molecular markers of early diagnosis or targeted intervention.

Our comprehensive immune profiling revealed distinct immunological landscapes between high-risk and low-risk LUAD patients, providing critical insights into disease progression and therapeutic response prediction. The paradoxical elevation of CD8 + T cells and plasma cells in high-risk tumors, despite their traditional anti-tumor roles, reflects dysfunctional immune activation rather than effective immunity. This phenomenon aligns with chronic antigen stimulation leading to T cell exhaustion, characterized by reduced effector function despite increased infiltration [40]. The concurrent enrichment of M0 macrophages and neutrophils in high-risk patients further supports an immunosuppressive microenvironment, as these cells polarize toward tumor-promoting phenotypes and facilitate cancer progression through NET formation and pro-angiogenic factor secretion [41–43]. Conversely, the depletion of gamma delta T cells and resting mast cells in high-risk tumors indicates compromised immune surveillance [44]. The positive correlation between all three prognostic genes and mast cell infiltration is noteworthy, as resting mast cells contribute to anti-tumor immunity through IFN-γ production and dendritic cell activation [45].

ESTIMATE analysis revealed significantly elevated stromal scores in high-risk patients, indicating extensive desmoplasia that creates physical barriers to immune infiltration. The lack of significant immune score differences emphasizes that functional immune states matter more than overall immune abundance. Most clinically relevant, low-risk patients demonstrated significantly lower TIDE and Exclusion scores, strongly predicting superior immunotherapy responses. This suggests prognostic risk stratification could serve as a predictive biomarker for immunotherapy patient selection, with low-risk LUAD patients representing optimal candidates for checkpoint inhibitors. The differential expression of immune checkpoint molecules (BTN2A2, BTNL9, CD47) in low-risk patients may reflect balanced immune regulation rather than overt suppression. In summary, high-risk LUAD is characterized by exhausted immune activation, stromal barriers, and T cell exclusion, whereas low-risk disease maintains functional immune homeostasis with greater immunotherapy sensitivity. These findings warrant prospective validation and suggest combination strategies targeting both prognostic pathways and immune checkpoints may benefit high-risk patients.

Drug prediction analysis shows that 8 small molecule drugs such as genistein, metronidazole, menadione, quercetin may have potential therapeutical effect for LUAD. Genistein is a phytoestrogen, and studies have shown it can exert anti-metastatic effects in lung cancer cells (A549) by downregulating the ERK1/2 and PI3K/Akt pathways [46]. Menadione (vitamin K3), a synthetic naphthoquinone derivative, generates reactive oxygen species that selectively damage cancer cells, induces apoptosis through mitochondrial dysfunction, and disrupts cellular bioenergetics by interfering with electron transport chain complexes [47]. Quercetin, a ubiquitous flavonoid, exhibits pleiotropic anti-cancer mechanisms including cell cycle arrest, apoptosis induction, angiogenesis suppression [48]. The binding energy of these drugs to key genes is low, indicating that they have good binding affinity. In the future, the therapeutic effect and mechanism of these drugs can be further verified through in vivo and in vitro experiments.

This study employed a multi-method integrated analytical strategy to elucidate the critical role of RAS signaling in LUAD pathogenesis at the transcriptomic level. Through systematic screening, we identified MAPK10, SHC3, and PLA2G12B as key molecular mediators regulating LUAD progression and predicted potential therapeutic compounds. These findings enhance our understanding of LUAD molecular mechanisms and provide valuable insights for molecular subtyping, precision therapy, and drug development. However, several limitations should be acknowledged. First, although the prognostic model was validated across three independent cohorts of different platforms and sample sizes, further multi-center validation incorporating larger cohorts from diverse geographic and ethnic populations would be valuable to confirm the generalizability of these findings across broader clinical settings. Second, our computational analyses lack experimental verification. The mechanistic roles of these key genes, pathways, and predicted drugs necessitate rigorous validation through in vitro and in vivo experiments to confirm their functional significance. Third, this investigation relies exclusively on transcriptomic data without integrating additional omics layers such as proteomics and metabolomics, which limits the comprehensiveness of our molecular characterization. Future research should incorporate emerging technologies such as spatial transcriptomics to investigate LUAD pathogenesis at higher resolution, dissecting spatial organization within the tumor microenvironment and capturing spatially-resolved cellular interactions that conventional bulk or single-cell analyses cannot resolve [49]. Additionally, we could leverage integrative GWAS platforms to establish genetic causal evidence for the identified prognostic genes via Mendelian randomization [50]. Such integrative approaches will provide a more robust theoretical foundation for precision medicine strategies in LUAD treatment.

## Electronic Supplementary Material

Below is the link to the electronic supplementary material.

Supplementary Material 1

## Data Availability

All data used in this study are publicly available. The LUAD-related transcriptomic datasets GSE31210 and GSE72094 were downloaded from the Gene Expression Omnibus (GEO) database (https://www.ncbi.nlm.nih.gov/geoprofiles/). The TCGA-LUAD dataset was downloaded from the TCGA database (https://portal.gdc.cancer.gov/). A total of 238 genes related to the RAS pathway were obtained from the KEGG database (https://www.genome.jp/kegg/). Additionally, protein-protein interaction (PPI) analysis was performed using the STRING database (https://cn.string-db.org/), gene set enrichment analysis (GSEA) was conducted using the MSigDB database (https://www.gsea-msigdb.org/gsea/msigdb), and drug prediction was carried out using the Connectivity Map (CMap) database (https://www.broadinstitute.org/connectivity-map-cmap).
