# Supplementary material for: RAS pathway activity subtypes identified by machine learning define prognostic and immune microenvironment characteristics in lung adenocarcinoma
Source: Discov Oncol. 2026 May 28;17:1085. doi: 10.1007/s12672-026-05282-9 (PMC13407407; doi:10.1007/s12672-026-05282-9)
Supplement: Supplementary file 1 — Supplementary Material 1 [file 12672_2026_5282_MOESM1_ESM.docx]

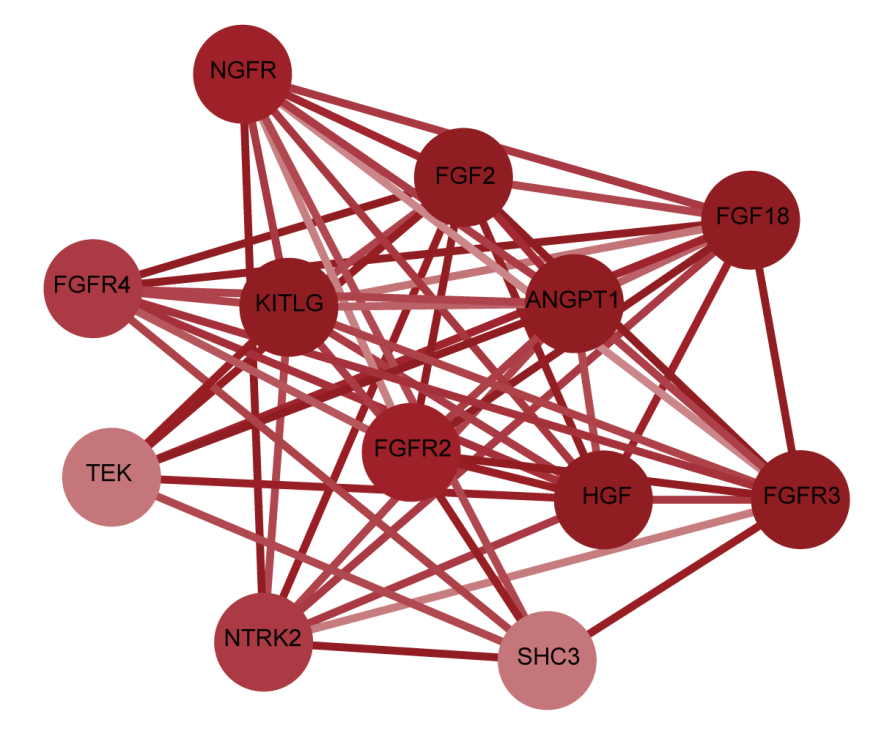


**Supplementary Figure 1 PPI Interaction Network Diagram**


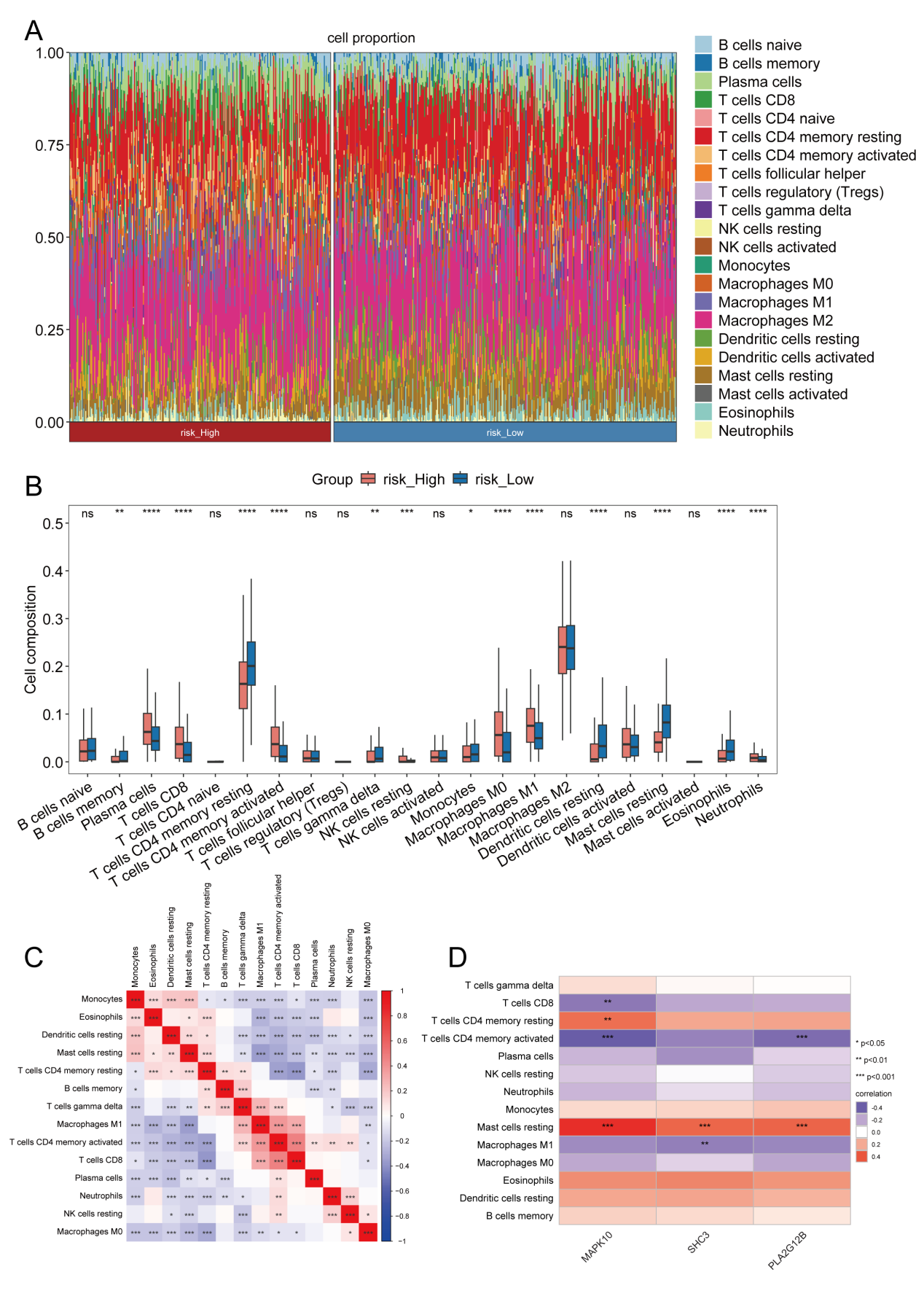


**Supplementary Figure 2. Analysis of the immune microenvironment**

(A) Immune cell distribution across risk groups. (B) Infiltration proportion comparisons. (C) Inter-cell correlation heatmap; color intensity indicates correlation strength (red: positive, blue: negative). (D) Prognostic gene-immune cell correlations.

Note: ns, no significant difference; *P < 0.05, **P < 0.01, ***P < 0.001, ****P < 0.0001.

**Supplementary Table 1. Binding free energies between key genes and small molecule drugs.**

| pert_iname | cell_iname | moa | raw_cs | fdr_q_nlog10 | targets |
| --- | --- | --- | --- | --- | --- |
| genistein | ASC | Tyrosine kinase inhibitor | -0.3117 | 0.0001 | SHC3 |
| metronidazole | U2OS | Bacterial DNA inhibitor | -0.4188 | 0.0001 | SHC3 |
| menadione | HA1E | Mitochondrial inhibitor\|Phosphatase inhibitor | -0.1863 | 0.0001 | MAPK10 |
| TG-101348 | HS578T | JAK inhibitor\|FLT3 inhibitor | -0.2364 | 0.0001 | MAPK10 |
| metformin | A549 | Insulin sensitizer | -0.2554 | 0.0001 | MAPK10 |
| JNK-9L | HS578T | JNK inhibitor | -0.2598 | 0.0001 | MAPK10 |
| HG-6-64-01 | A549 | RAF inhibitor | -0.2711 | 0.0001 | MAPK10 |
| menadione | HA1E | Mitochondrial inhibitor\|Phosphatase inhibitor | -0.1863 | 0.0001 | PLA2G12B |
| quercetin | YAPC | Polar auxin transport inhibitor | -0.2856 | 0.0001 | PLA2G12B |
